# Area-level socioeconomic variables associated with territorial disparities in tuberculosis notification rates in metropolitan France: a Bayesian ecological analysis

**DOI:** 10.1186/s40249-025-01354-0

**Published:** 2025-09-19

**Authors:** Camille Pelat, Anne Bernadou, Philippe Fraisse, Cyrille Delpierre, Yousra Kherabi, Jean-Paul Guthmann, Stéphanie Vandentorren

**Affiliations:** 1 Santé publique France, The national public health agency, Saint-Maurice, France; 2 Santé publique France, The national public health agency–Nouvelle-Aquitaine, Bordeaux, France; 3 French National Network of Tuberculosis Control Centers, Strasbourg, France; 4 Group for research and teaching in pneumo-infectiology, French-speaking pneumology society, Paris, France; 5 Specialized Commission on Health Care System and Patient Safety, High Council of Public Health, Paris, France; 6 Center for Epidemiology and Research in POPulation Health, National Institute of Health and Medical Research, Université de Toulouse, Toulouse, France; 7 Infectious and Tropical Diseases Department, Bichat-Claude Bernard Hospital, Assistance Publique-Hôpitaux de Paris, Université Paris Cité, Paris, France; 8 Infection, Antimicrobials, Modelling and Evolution research unit, National Institute of Health and Medical Research, Université Paris Cité, Paris, France; 9 Population Health Translational Research team, Bordeaux Population Health Research Center, National Institute of Health and Medical Research, Bordeaux, France; 10 University of Bordeaux, Bordeaux, France

**Keywords:** Tuberculosis, Health inequality, Ecological study, Area-level socioeconomic variables

## Abstract

**Background:**

Although France is considered a low tuberculosis (TB) incidence country, TB remains a significant public health issue in certain high-risk groups and geographic areas, potentially linked to socioeconomic determinants. This study aims to assess the associations between TB notification rates and area-level socioeconomic variables in metropolitan France.

**Methods:**

We conducted an ecological spatial study using TB cases reported to the French national surveillance system from 2008 to 2019. Using Bayesian Poisson regression, we modeled TB case counts at the ZIP code level. Standardized notification rates were estimated through indirect standardization by age, sex, immigration status, and housing type. The model included ZIP code level socioeconomic variables and a spatial random effect to account for spatial autocorrelation and residual variations in notification rates, which may relate to territorial disparities in reporting completeness.

**Results:**

The study included 55,330 reported TB cases across 4478 of 5534 ZIP codes in metropolitan France. All tested socioeconomic variables showed varying associations with TB. In the multivariable model, an increase in population density from ‘Low’ to ‘High’ was associated with a 30% increase [95% credible interval (CrI): 21%, 38%] in standardized TB notification rates. An increase from the first to the ninth decile in the unemployment rate among those aged 15–64 was associated with a 28% increase (95% CrI: 19%, 37%). Similarly, an increase in the proportion of overcrowded households was associated with a 19% increase (95% CrI: 11%, 28%). Conversely, an increase in median household income was associated with a 7% decrease (95% CrI: 1%, 11%).

**Conclusions:**

Our findings suggest that TB notification rates are independently associated with material deprivation, such as unemployment and low income, as well as crowded settings, including overcrowded households and densely populated areas. Enhancing TB control in metropolitan France could involve targeted outreach programs for screening and treatment in materially deprived areas, characterized by high unemployment rates and low median incomes, and adopting a ‘Health in All Policies’ approach to address urban and household crowding.

**Graphical Abstract:**

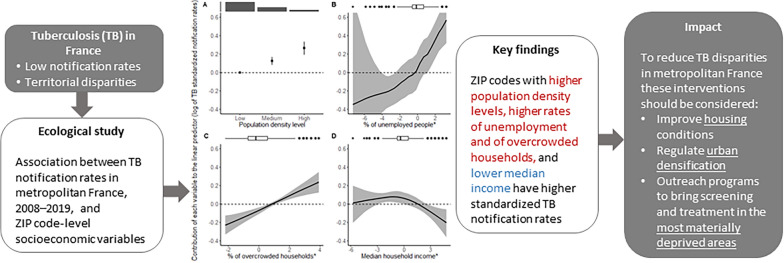

**Supplementary Information:**

The online version contains supplementary material available at 10.1186/s40249-025-01354-0.

## Background

Tuberculosis (TB) is an airborne, curable, and preventable disease caused by the bacterium *Mycobacterium tuberculosis*. According to the World Health Organization (WHO), an estimated 10.8 million new cases of TB occurred worldwide in 2023, compared to 10.7 million cases in 2022 [1]. In the same year, TB likely returned to being the world's leading cause of death from a single infectious agent, surpassing COVID-19, with 1.25 million deaths (compared to 1.32 million in 2022). The prevalence and incidence of TB vary both between and within countries. In 2023, the WHO Southeast Asia and African regions reported the highest number of new TB cases, with the WHO European Region recording an estimated 225,000 cases [2].

In France, TB has been a notifiable disease since 1964. Over the past three decades, notification data indicate an average annual decline in incidence of 1.7%, with a noticeable rebound in 2023 (+ 15%) [3]. France is considered a low-incidence country, with 4866 notified TB cases in 2023, corresponding to a rate of 7.1 cases per 100,000 people. However, there is an uneven distribution across social groups and regions [3]. TB predominantly affects individuals experiencing homelessness (69 cases per 100,000), prisoners (49 cases per 100,000), and foreign-born individuals (37 cases per 100,000) (2023 data) [3]. The Île-de-France administrative region, which includes Greater Paris, and the two overseas regions of French Guiana and Mayotte had the highest notification rates in 2023, all exceeding 10 cases per 100,000 inhabitants. Moreover, a study on data from 2013–2018 in Île-de-France reported intra-regional disparities in notification rates, which correlated with variations in poverty rates [4].

Multiple ecological studies in low-, middle- and high-income countries have highlighted associations between the spatial distribution of TB and area-level socioeconomic variables, such as median household income, unemployment rates, undernourishment, and poor housing conditions [5–8]. Other studies have investigated the mechanisms through which social determinants of health increase an individual’s risk of acquiring, developing, and transmitting TB [9]. For example, individuals facing socioeconomic deprivation are at greater risk of contact with infectious TB carriers and are more likely to live and work in crowded and poorly ventilated places. They are also more likely to experience frequent risk factors for TB infection after contact with the bacillus and/or progression from latent to active infection, including smoking, alcohol abuse, comorbidities such as HIV or diabetes, and malnutrition. Furthermore, they face barriers to timely TB diagnosis and completion of a full TB treatment course, which prolongs their symptomatic infectious period and increases the risk of transmission to others within their community [10–12].

To date, no nationwide ecological study has assessed the association between TB notification rates and socioeconomic territorial inequalities in France. The goal of this study is to identify area-level socioeconomic variables associated with TB notification rates in metropolitan France. This evidence could help tailor TB control policies—for example, by strengthening actions in the most socially vulnerable areas—following a proportionate universalism approach [13].

## Methods

### Design, period and study area

We conducted a retrospective ecological study in metropolitan France for the period of 2008–2019. The data were aggregated into two six-year periods and at a quasi-ZIP code geographic level. More precisely, we chose a specific geographic division (called the PMSI21 code), which corresponds to existing ZIP codes with ≥ 1000 inhabitants in 2021 and to aggregations of neighboring ZIP codes with fewer than 1000 inhabitants [14]. For clarity and consistency, this territorial division is hereafter designated the “ZIP code”.

### Data collection

#### TB cases

TB cases reported in metropolitan France from 2008 to 2019 were extracted from the national notifiable disease surveillance system for analysis. All forms of TB, including both pulmonary and extrapulmonary cases, were retained in the analysis. The case data included the ZIP code of the person’s usual place of residence, age, sex, countries of birth of both the individual and their parents, and whether the person lived in communal housing (yes/no). If the person lived in communal housing, the type of communal housing was specified as prison, residence for elderly people, collective shelter, or other communal housing. A single, simplified ‘housing type’ variable was constructed from these two variables: it was categorized as ‘individual housing’ when cases indicated not living in communal housing, ‘prison’ when the type of communal housing was ‘prison’, and ‘other communal housing’ otherwise. Cases with a missing or incorrect ZIP code were excluded from the analysis.

We classified cases as ‘immigrant’ or ‘native French’, adhering as closely as possible to the definition of immigrants by the French National Institute of Statistics and Economic Studies (INSEE): “An immigrant is a person born as a foreigner abroad and residing in France” [15]. Given that we only had data on the country of birth, individuals were classified as ‘immigrants’ if they were born outside of France and either both parents were foreign-born or data on parental countries of birth were unavailable. All other cases were classified as ‘native French’.

### Socioeconomic area-level data

We examined the associations at the ZIP code level between TB notification rates and multiple socioeconomic variables provided by INSEE. These variables were selected based on documented risk factors for TB in low-endemic countries from the literature. Initially, we analyzed the following variables separately: median household income per consumption unit, the proportion of high school graduates in the unschooled population aged 15 years and older, the proportion of manual workers in the active population aged 15–64 years, and the unemployment rate among the active population aged 15–64 years. We then estimated the associations between TB notification rates and a social deprivation indicator that combines these four variables, known as the French Deprivation Index (FDep) [16]. Additionally, we measured the associations between TB notification rates and the proportion of overcrowded households (see definition in the Supplementary Information) [17], as well as the population density level (Low, Medium, or High) [18].

For the first period, continuous variables were derived from municipal or sub-municipal datasets from the 2009 or 2010 censuses, whereas for the second period, they were obtained from the 2015 and 2016 censuses (see Supplementary Table 1). We primarily utilized population-weighted means to calculate ZIP code-level values for most continuous variables. However, for the median household income per consumption unit, we employed population-weighted medians. The population density level of municipalities was consistent across both periods, as data was solely available from the 2010 census. To establish ZIP code population density levels, we computed the cumulative number of inhabitants in 2010 for each density level within the municipalities corresponding to the same ZIP code [19].

### Statistical analysis

#### Standardization

We calculated the notified TB case counts in metropolitan France across all strata defined by each of the two studied periods (2008–2013 and 2014–2019), age group (0–14, 15–24, 25–44, 45–64, and 65 years or older), sex (male, female), immigration status (immigrant, native), and housing category (individual housing, prison, other communal housing). Missing values were present in the last four variables, and were imputed using the k-nearest neighbors (kNN) imputation method implemented in the R package VIM [20]; see the supplementary file for more details. The reference notification rate was calculated in each stratum by dividing the number of notified TB cases by the corresponding population in metropolitan France, derived from the 2010 and 2016 censuses (ad hoc files provided by INSEE).

Finally, the reference notification rates were multiplied by the population of each stratum in each ZIP code (using 2010 and 2016 as reference years for the periods 2008–2013 and 2014–2019, respectively) and summed to obtain the expected number of TB cases by ZIP code and period.

#### Modeling

The number of TB cases notified in ZIP code *i* in period *t,* ${n}_{i,t}$, was assumed to follow a Poisson distribution of mean ${E}_{i,t}{\lambda }_{i}$, with ${E}_{i,t}$ being the expected number of TB cases notified in ZIP code *i* and period *t* and ${\lambda }_{i,t}$ a relative risk known as the standardized notification rate (SNR).

$${n}_{i,t}\sim Poisson\left({{\lambda }_{i,t}E}_{i,t}\right)$$

We modeled ${\lambda }_{i,t}$ via ${\alpha }_{0}$, a constant, ${b}_{i}$, a spatial random effect, and explanatory variables. The median household income per consumption unit, the proportion of high school graduates in the unschooled population aged ≥ 15 years, the proportion of manual workers in the active population aged 15–64 years, the proportion of unemployment rate among the active population aged 15–64 years, and the proportion of overcrowded households were log-transformed to reduce distribution skewness. All the continuous explanatory variables were then standardized by period and entered into the model with smoothed functions:

$$\textrm{log}\left( {\lambda_{i,t} } \right)\; = \,\alpha_{0} + b_{i} + f_{k} \left( {X_{i,t}^{k} } \right),$$

Where ${X}_{i,t}^{k}$ is the standardized measure of the ${k}^{th}$ explanatory variable in ZIP code *i* and period *t* and where ${f}_{k}$ is an order 2 random walk function. We opted to standardize the explanatory variables by period due to our primary interest in their spatial contrasts within each period. Additionally, changes in the data collection method occurred between 2010 and 2016, particularly concerning the median household income per consumption unit.

We modeled the effect of the population density level with dummy variables:

$$\textrm{log}\left( {\lambda_{i,t} } \right)\; = \,\alpha_{0} + b_{i} + \alpha_{1} I_{i}^{Medium} + \alpha_{2} I_{i}^{High} ,$$

Where ${I}_{i}^{Medium}$ (respectively$, {I}_{i}^{High}$) was equal to 1 if the population density level was ‘Medium’ (respectively, ‘High’) in ZIP code *i*, 0 otherwise.

The spatial random effect, ${b}_{i}$, was employed to model spatial autocorrelation and residual variations in the standardized TB notification rates. These residual variations could be linked, for example, to territorial disparities in the completeness of the reporting system. ${b}_{i}$ was assigned a BYM2 distribution [21], which includes a spatially structured component, ${u}_{i}$, following a standardized intrinsic conditional autoregressive model, and a random component, ${v}_{i}$, following a standard normal distribution: ${b}_{i}=\frac{1}{\sqrt{{\tau }_{b}}}\left(\sqrt{1-\phi }{v}_{i}+\sqrt{\phi }{u}_{i}\right)$, with $\frac{1}{{\tau }_{b}}$, the marginal variance of ${b}_{i}$.

We utilized the deviance information criterion (DIC) to identify the variables most strongly associated with TB notification rates; lower DIC values indicated a better fit. We subsequently tested whether the association between socioeconomic variables and TB notification rates changed between periods by adding a time × period interaction to each univariable model. In practice, we modeled the nonlinear effect of the continuous variables using a second-order random walk model specific to each period, with both models sharing the same hyperparameters. The interaction effect of the population density level with the period was modeled using a factor × factor interaction term. We assessed the relevance of the interaction terms by graphically examining the overlap of the period-specific effects (and their credible intervals).

We ultimately developed a multivariable model incorporating variables with the lowest DIC values and minimal correlation to avoid multicollinearity:

$$\textrm{log}\left({\lambda }_{i,t}\right)={\alpha }_{0}+{b}_{i}+{f}_{1}\left({X}_{i,t}^{1} \right)+\dots +{f}_{K}({X}_{i,t}^{K})+{\alpha }_{1}{I}_{i}^{Medium}+{\alpha }_{2}{I}_{i}^{High}$$

### Associations with explanatory variables

Based on the multivariable model, we examined the shape of the association between each explanatory variable and the logarithm of the SNR (log-SNR, the linear predictor of the models). To that effect, we plotted the partial contribution of each variable ${X}^{k}$ to the linear predictor, ${\widehat{f}}_{k}\left({X}_{i,t}^{k}\right)$, along with its 95% credible interval (95% CrI). This approach allowed us to identify which explanatory variables contributed significantly to the predictions.

To assess the extent of the SNR variation across the distribution of each explanatory variable, we calculated the ratio of the SNRs at their 10^th^ and 90^th^ percentiles and labeled it the “inter-decile standardized rate ratio” (IdRR). In this, we adopted an approach proposed by Larsen et al. [22, 23] in the context of multilevel logistic regression models, where they developed the “interval odds ratio” to reflect the variation in the odds ratio due to random effects in the linear predictor. Similarly, Chaix et al. developed the “interquartile spatial odds ratio”, defined as the odds ratio between an individual residing in a location in the first quartile and one from a location in the fourth quartile of spatial risk.

### Sensitivity analyses

To assess the robustness of our findings to the chosen distribution of the spatial random effect, we replaced the BYM2 distribution with the Leroux distribution [24] and reran the model without explanatory variables as well as the multivariable model. Like the BYM2 model, the Leroux model comprises both structured and unstructured components but differs in their parameterization (see supplementary file section 4.2.1 for further details).

We also evaluated the sensitivity of the analysis to the chosen imputation method (kNN) by imputing missing values using two alternative approaches. Initially, we utilized the random forest imputation algorithm from the R package missForest [25]. Subsequently, we proportionally distributed the missing values across strata defined by sex, age group, immigration status, and housing type, utilizing information from cases with similar characteristics. Further details are provided in supplementary file section 5.1.

All statistical analyses were performed using R version 4.2.3 [26] and INLA version 22.12.16 [27, 28].

## Results

Our study population consisted of 55,330 reported TB patients: 28,410 patients were reported during 2008–2013 and 26,920 during 2014–2019. These cases were distributed across 4478 out of 5534 ZIP codes in metropolitan France. Among them, 72% had pulmonary TB. Table 1 further illustrates the distribution of TB cases by age, sex, immigration status, pulmonary form (yes/no), and type of housing, stratified by period. Missing data primarily impacted the constructed variables ‘type of housing’ (13.4%) and ‘immigration status’ (7.1%), with only marginal effects on the variables sex (0.6%) and age (0.1%). Supplementary Table 2 provides a description of the ZIP code-level explanatory variables, stratified by period, while the distributions of these explanatory variables, both raw and log-transformed, are displayed in Supplementary Figure 1.

**Table 1.** Characteristics of the studied sample of TB patients (*n* = 55,330), extracted from the national notifiable disease surveillance system, metropolitan France, 2008–2013 and 2014–2019

	[ALL]	2008–2013	2014–2019	*P* value	N^$^
Age, Median [25^th^ percentile;75^th^ percentile]	41 [28;62]	43 [29;64]	39 [27;59]	< 0.001*	55,288
Male, *n* (%)	33,934 (62%)	16,892 (60%)	17,042 (64%)	< 0.001^¥^	54,982
Immigrant, *n* (%)	29,512 (57%)	13,828 (53%)	15,684 (62%)	< 0.001^¥^	51,417
Pulmonary TB, *n* (%)	36,215 (72%)	20,469 (72%)	15,746 (71%)	0.009^¥^	50,589
Type of housing, *n* (%):				< 0.001^¥^	47,921
Individual housing	40,547 (85%)	21,395 (86%)	19,152 (83%)		
Prison	564 (1%)	272 (1%)	292 (1%)		
Other communal housing	6810 (14%)	3099 (13%)	3711 (16%)		

^*^Kruskall-Wallis test

^¥^Chi-square test

^$^Number of nonmissing observations for each variable, among the 55,330 cases

Table created with the R package compareGroups [48]

### Associations of area-level socioeconomic variables with TB in univariable models

**Fig. 1.**
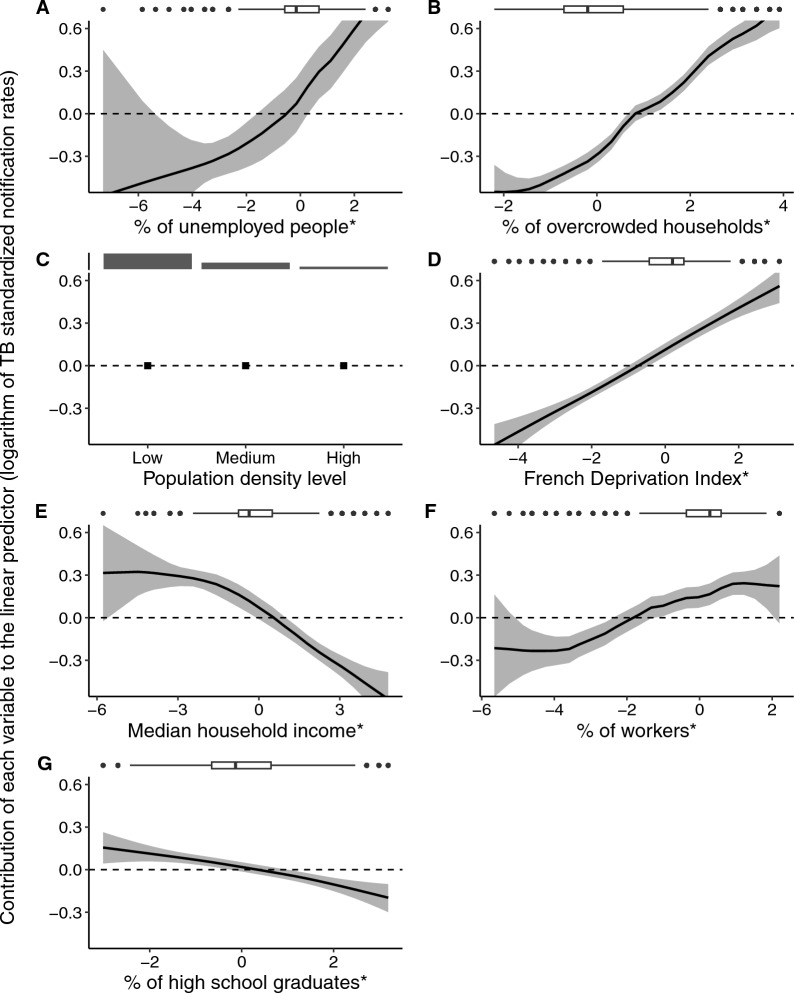
Associations between ZIP code-level socioeconomic variables and TB standardized notification rates via univariable models. The models are sorted by decreasing goodness-of-fit (increasing deviance information criterion). The models include a spatial random effect modeled with a BYM2 distribution and one of the following variables: (**A**) the unemployment rate among the active population aged 15–64 years; (**B**) the proportion of overcrowded households; (**C**) the population density level; (**D**) the French Deprivation Index (a social deprivation indicator combining four variables); (**E**) the median household income per consumption unit; (**F**) the proportion of manual workers in the active population aged 15–64 years; and (**G**) the proportion of high school graduates in the unschooled population aged ≥15 years. The shaded areas represent 95% credible intervals. *These variables were log-transformed (except for the French Deprivation Index) and standardized by period before being entered into the models. Boxplots and barplots at the top illustrate the variable distributions

The shape of the association between each explanatory variable and the SNR is illustrated in Figure 1. Among the univariable models, that incorporating the unemployment rate among the active population aged 15–64 years exhibited the lowest DIC value, indicating the best fit. The subsequent models in terms of DIC performance included those with the proportion of overcrowded households, the population density level, the FDep, and the median income per consumption unit (see Table 2). The population density level and the proportion of overcrowded households yielded the highest IdRR [1.74, 95% CrI (1.62, 1.86)] and [1.74, 95% CrI (1.61, 1.89)], respectively. Specifically, a transition from the ‘Low’ population density level, which comprises 63% of ZIP codes, to the ‘High’ population density level, encompassing 10% of ZIP codes, was associated with a 74% increase in the standardized TB notification rate. Similarly, a transition from the first decile to the ninth decile of the proportion of overcrowded households was associated with the same magnitude of increase.

**Table 2.** Results of the univariable models associating TB notification rates with ZIP code-level socioeconomic variables

Univariable model	DIC	IdRR (95% Crl)
Unemployment rate among the active population aged 15–64 years	36,023	1.56 (1.47, 1.64)
Proportion of overcrowded households	36,109	1.74 (1.61, 1.89)
Population density level	36,117	1.74 (1.62, 1.86)
French Deprivation Index (FDep)	36,118	1.49 (1.38, 1.58)
Median household income per consumption unit	36,171	0.73 (0.69, 0.76)
Proportion of manual workers in the active population aged 15–64 years	36,187	1.18 (1.12, 1.25)
Proportion of high school graduates in the unschooled population aged ≥ 15 years	36,208	0.90 (0.85, 0.94)
Empty model	36,230	

The “empty” model comprises solely the spatial random effect. The inter-decile standardized rate ratio (IdRR) indicates by how much the standardized notification rates are multiplied when each explanatory variable value increases from its first decile to its ninth decile. Data source: national notifiable disease surveillance system, metropolitan France, 2008–2019. DIC: deviance information criterion, Crl: credible interval

The population density level was retained in the multivariable model, along with the continuous variables that best fit the TB notification data, as indicated by the lowest DIC, while ensuring minimal collinearity (assessed via the correlation matrix presented in Supplementary Figure 2). The FDep was excluded because its construction was partially based on the proportion of unemployed individuals, which was among the selected variables. The final set of continuous explanatory variables included: (i) the unemployment rate among the active population aged 15–64 years, (ii) the proportion of overcrowded households, and (iii) the median household income per consumption unit.

**Fig. 2.**
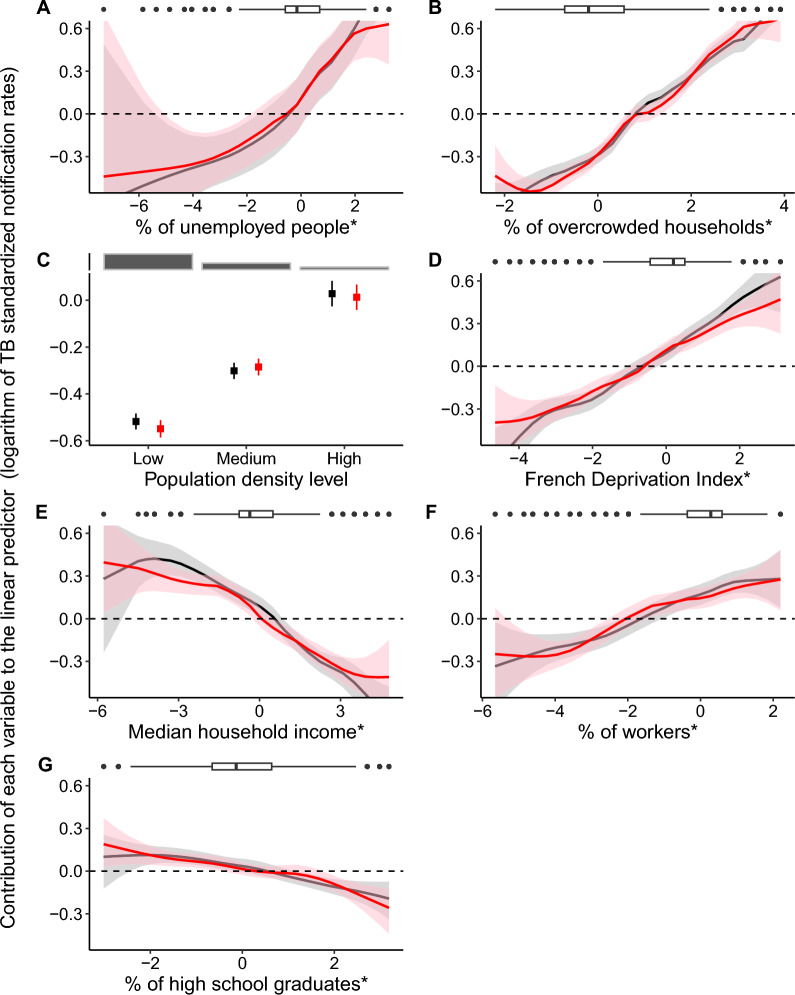
Associations between ZIP code-level socioeconomic variables and TB standardized notification rates by period, via univariable models. Black: the 2008–2013 period; red: the 2014–2019 period. The models include a spatial random effect modeled with a BYM2 distribution and one of the following variables, in interaction with the period: (**A**) the unemployment rate among the active population aged 15–64 years; (**B**) the proportion of overcrowded households; (**C**) the population density level; (**D**) the French Deprivation Index (a social deprivation indicator combining four variables); (**E**) the median household income per consumption unit; (**F**) the proportion of manual workers in the active population aged 15–64 years; and (**G**) the proportion of high school graduates in the unschooled population aged ≥ 15 years. The shaded areas represent 95% credible intervals. *These variables were log-transformed (except for the French Deprivation Index) and standardized by period before being entered into the models. Boxplots and barplots at the top illustrate the variable distributions

Figure 2 shows that the effect of the explanatory variables remains consistent across periods, indicating that it was unnecessary to retain the interaction terms in the multivariable model.

### Assessment of area-level socioeconomic variables in a multivariable context

**Fig. 3.**
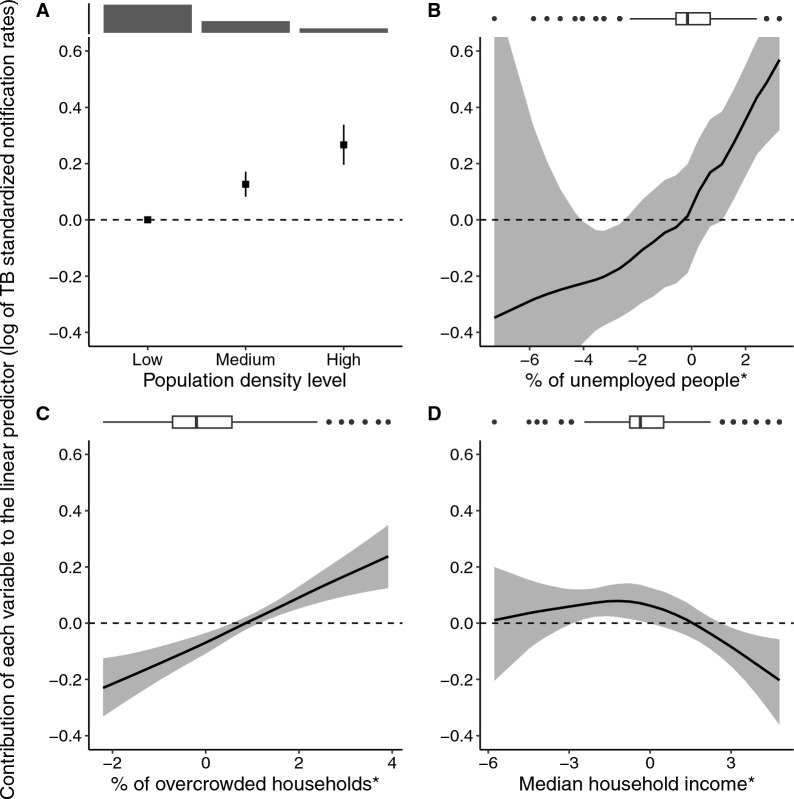
Associations between ZIP code-level socioeconomic variables and TB standardized notification rates according to the multivariable model. The variables are sorted by decreasing inter-decile standardized rate ratio. The model includes a spatial random effect modeled with a BYM2 distribution and the following four variables: (**A**) the population density level; (**B**) the unemployment rate among the active population aged 15–64 years; (**C**) the proportion of overcrowded households; and (**D**) the median household income per consumption unit. The shaded areas represent 95% credible intervals. *These variables were log-transformed and standardized by period before being entered into the model. Boxplots and barplots at the top illustrate the variable distributions

As illustrated in Figure 3, the multivariable model reveals that the TB notification rate increased with the unemployment rate among the active population aged 15–64 years, the proportion of overcrowded households, and the population density level, whereas it decreased with the median household income per consumption unit. Table 3 presents the IdRR for each socioeconomic variable, indicating that a change in the population density level from ‘Low’ to ‘High’ was associated with a 30% increase (95% CrI: 21%, 38%) in standardized TB notification rates. Additionally, a change from the first to the ninth decile in the unemployment rate among the active population aged 15–64 years was associated with a 28% increase (95% CrI: 19%, 37%). Similarly, a change from the first to the ninth decile in the proportion of overcrowded households was associated with a 19% increase (95% CrI: 11%, 28%). Conversely, a change from the first to the ninth decile in the median household income per consumption unit was associated with a 7% decrease (95% CrI: 1%, 11%). Maps of the spatial random effects in the model without explanatory variables and the multivariable model are presented in Supplementary Figure 4.

**Table 3.** Results of the multivariable model associating TB notification rates with ZIP code-level socioeconomic variables

Variable	IdRR (95% CrI)
Population density level	1.30 (1.21, 1.38)
Unemployment rate among the active population aged 15–64 years	1.28 (1.19, 1.37)
Proportion of overcrowded households	1.19 (1.11, 1.28)
Median household income per consumption unit	0.93 (0.89, 0.99)

The inter-decile standardized rate ratio (IdRR) indicates by how much the standardized notification rates are multiplied when each explanatory variable value increases from its first decile to its ninth decile. Data source: national notifiable disease surveillance system, metropolitan France, 2008–2019. CrI: credible interval

### Sensitivity analyses

#### Alternative spatial model

As detailed in supplementary file section 4.2, the spatial random effects derived from the BYM2 model (central analysis) and the Leroux model (sensitivity analysis) exhibited strong similarities, both in the maps and the histograms. The effects of the explanatory variables were also highly comparable between the two models, both graphically and as quantified by the IdRRs. Furthermore, the BYM2 model and the Leroux model demonstrated comparable goodness-of-fit statistics.

#### Alternative imputation methods

The results of the multivariable model, based on data imputed using two alternative methods, are compared with those of the reference model in supplementary file section 5.2. In summary, all three models yielded similar results for the standardized notification rates and the spatial random effects. Additionally, the effects of each explanatory variable on the predictor were equivalent in terms of shape and magnitude, as were the IdRRs, which summarize these effects.

## Discussion

In this study, we examined the relationships between ZIP code-level socioeconomic variables and TB notification rates, standardized by age, sex, immigration status and type of housing, in metropolitan France between 2008 and 2019. The selected variables were the median household income (per consumption unit), the proportion of high school graduates in the unschooled population aged ≥15 years, the proportion of manual workers in the active population aged 15–64 years, the unemployment rate among the active population aged 15–64 years, a composite indicator of the preceding four variables (the social deprivation index, FDep), the proportion of overcrowded households, and the population density level. In the univariable analyses, all of these variables were associated with TB—though to different extents. TB notification rates increased with population density level, proportion of overcrowded households, unemployed individuals and workers, and FDep. It decreased with the median household income and the proportion of high school graduates. By testing interaction terms, we confirmed that the associations between all investigated socioeconomic area-level variables and standardized TB notification rates remained consistent between the two study periods (2008–2013 and 2014–2019). This consistency indicated that the inclusion of an interaction term was unnecessary.

Finally, a multivariable model was built incorporating the variables most associated with TB (as defined by the lowest DIC values) and with minimal correlation to avoid multicollinearity. These included: population density, the unemployment rate among the active population aged 15–64 years, the proportion of overcrowded households, and the median household income per consumption unit. We found that in a multivariable context, these four variables were independently and significantly associated with standardized TB notification rates. These findings are consistent with findings in Europe [5, 29] and in the United States, South Africa, China, and Brazil [8, 30–32].

### Deprivation index vs. its components

In the univariable analyses, the FDep—a composite deprivation index—showed a weaker association with TB than the unemployment rate among the active population aged 15–64 years, which is one of its four components. In addition, the median household income per consumption unit, the proportion of manual workers in the active population aged 15–64 years and the proportion of high school graduates in the unschooled population aged ≥ 15 years were less strongly associated with TB than the FDep. This suggests that some dimensions of the FDep are more relevant for TB transmission in France than others are: directly using the most relevant variables in the analysis can provide more accurate and more directly interpretable results. A similar conclusion was put forward by Apolinario et al. in an ecological study conducted in Portugal: the European deprivation index was less strongly associated with TB incidence than two of the variables used to build this index—the unemployment rate and that of manual workers [5].

With respect to France, the proportion of manual workers was not among the best predictors of standardized TB notification rates. The unemployment rate and low median household income, which are markers of material deprivation, were the FDep dimensions most associated with TB. These variables have already been reported to be associated with TB in several other studies [5, 7, 8, 33, 34].

### Overcrowding

We also found that areas with a high proportion of overcrowded households were associated with higher standardized TB notification rates. This result is in line with those of several studies conducted in rich or poor settings at the country or city level [35–38]. Overcrowding may increase the frequency of close contacts, promoting TB transmission between household members. It may also be a marker of other poor housing conditions linked to a greater transmission risk of airborne pathogens, for example, inadequate ventilation or poor indoor air quality [39].

Finally, ZIP codes with high population density levels were associated with higher standardized TB notification rates. Previous studies have highlighted the associations between TB risk and urban, densely populated settings or crowded gathering places [5, 32, 40–42]. These results are consistent with dense settings increasing the opportunities for contact with TB carriers outside the household.

### Sensitivity analyses

We confirmed that the model outputs were robust to the choice of the imputation method by testing two alternative imputation techniques. Additionally, we verified that an alternative specification of the spatial random effect—using the Leroux model—produced results consistent with those obtained from the BYM2 model. This consistency was theoretically anticipated, as demonstrated by Riebler et al. [21]. Furthermore, Riebler et al. noted that none of the alternatives to the BYM2 model were appropriately scaled, which complicates the selection of a prior distribution for their precision parameter. This ambiguity reinforces the BYM2 model as the preferred choice.

### Strengths and limitations

This study has several notable strengths. First, our analysis was based on a substantial sample size, encompassing over 55,000 TB cases across metropolitan France. Additionally, we utilized age, sex, immigration status, and housing type—stratified over two six-year periods—to calculate reference TB notification rates. This methodological approach ensured that the standardized notification rates at the ZIP code level were not influenced by spatial and temporal variations in these four population characteristics. Consequently, our findings on the associations between standardized TB rates and socioeconomic area-level variables should be interpreted as applicable to individuals with the same sex, age class, immigration status, and housing type. To our knowledge, this represents the first instance in which immigration status and housing type have been incorporated into the computation of reference TB notification rates.

Our study also has limitations. First, the definition of immigration status for TB patients, based solely on the country of birth of patients and their parents, does not perfectly align with that published by INSEE for the population. Therefore, there may have been instances of misclassification, where some immigrants were incorrectly identified as native and some natives as immigrants. Second, a study estimated that only 73% of pulmonary TB cases were reported to the French TB notifiable disease surveillance system in 2010, with substantial inter-regional variation (from 45.5% in Auvergne to 99.5% in Brittany) and intra-regional heterogeneity [43]. This variability can be attributed to the structure of the national notifiable disease surveillance system for TB in France. In this system, each Regional Health Agency is responsible for collecting TB notifications before transmitting them to the national system. Consequently, the rigor with which these agencies maintain reporting completeness may vary. Through the use of a spatially structured random effect in the model, we have accounted for smoothed territorial differences in standardized TB notification rates, which encompass both unexplained differences in true incidence rates and differences in reporting completeness. Our hypothesis was that these “smooth” variations occurred on a scale sufficiently different from that of local contrasts in socioeconomic variables (i.e., at the regional level versus the ZIP code level), and that the two processes were independent, so that the model could distinguish the explanatory variable effects from the spatial random effects. The maps of the spatial random effects presented in Supplementary Figure 4 allowed us to confirm that this hypothesis was valid.

A second limitation is the decision to include data only until 2019, which prevented the inclusion of most recent patient information. This decision was made because of the observed decline in reported TB cases in France during the COVID-19 pandemic, as access to essential TB services, particularly diagnostics, was significantly disrupted in 2020. This decline was documented by the WHO for all regions of the world, including Europe [44]. A gradual return to pre-pandemic levels was observed in 2021, both in France [45] and internationally [44].

A third limitation is that we did not use multiple imputation techniques to address the missing values in the case characteristics used for stratifying the expected values. Consequently, the uncertainty in the calculation of the expected values is not considered in the results. Nevertheless, we verified with alternative imputation methods that our results remained robust to changes in imputation methods.

## Conclusions

Our analysis demonstrated that standardized TB notification rates at the ZIP code level in metropolitan France were independently and significantly associated with population density, the unemployment rate among the active population aged 15–64 years, the proportion of overcrowded households, and the median household income per consumption unit. While the ecological regression design of our study precludes the establishment of causation, these results are consistent with previous evidence and highlight the key role that poor housing conditions, high population density and material deprivation play in TB transmission in France. French public health authorities could use this argument to advocate for improved housing policies and regulated urban densification to mitigate TB transmission, following the “Health in All Policies” approach proposed by the WHO [46] and the French National Health Strategy 2023**–**2033 [47]. Finally, outreach programs could bring screening and treatment to the populations at the highest risk of TB, particularly those residing in areas characterized by high unemployment and poverty. The French National Health Strategy 2023**–**2033 acknowledges the need for such programs to reduce growing health inequalities [47].

## Supplementary Information

Supplementary Material 1

## Data Availability

The R scripts are provided in the GitHub repository: https://github.com/cpelat/TB-ContextualFactors-FR. The datasets of the contextual explanatory variables and the ZIP code-level observed and expected TB case counts, stratified by period (2008–2013 and 2014–2019), are also included. The raw individual data of TB patients used for the study were collected by Santé Publique France, the French national public health agency, through the national notifiable disease surveillance system. This was done in the application of a law (article L. 3113-1 of the "Code de la santé publique"—French Public Health Code) and a decree (article R3113-1 et seq. of the French Public Health Code), which require medical doctors and directors of medical biology laboratories to transmit to Santé Publique France pseudonymized data relating to their patients diagnosed with a disease listed by a decree (article D 3113-9 of the French Public Health Code). This list includes tuberculosis. These data are personal health data covered by professional confidentiality (R3113-6 French Public Health Code). In accordance with French legislation on the protection of personal data, they cannot be shared without the authorization of the French data protection authority (Cnil).

## Abbreviations

BYM2: Modified Besag-York-Mollié model
CrI: Credible interval
FDep: French Deprivation Index
IdRR: Inter-decile standardized rate ratio
TB: Tuberculosis
