# Supplementary material for: Area-level socioeconomic variables associated with territorial disparities in tuberculosis notification rates in metropolitan France: a Bayesian ecological analysis
Source: Infect Dis Poverty. 2025 Sep 19;14:94. doi: 10.1186/s40249-025-01354-0 (PMC12447623; doi:10.1186/s40249-025-01354-0)
Supplement: Supplementary file 1 — Supplementary Material 1 [file 40249_2025_1354_MOESM1_ESM.docx]

Supplementary information

# Data sources for the contextual variables

Table 1. Data sources for each contextual covariate, by study period

| **Variable** | **2008–2013 period** | **2014–2019 period** |
| --- | --- | --- |
| Median household income per consumption unit | Source: 2009 census data  Date downloaded: 2023-02-21  One file contained the FDep 2009 + its 4 components + the population size, at the sub-communal level: https://geo.data.gouv.fr/fr/datasets/9c6009a2bb10c4d69a15d399def4770b038be18a  We completed this file with two other files to be able to aggregate all variable values at the ZIP code levels:   - File “Diplomas - Training in 2009”, census data at the sub-communal level, produced by INSEE: contains the number of unschooled persons aged ≥15 years: https://www.insee.fr/fr/statistiques/2028257 - File “Resident activity in 2009” ”, census data at the sub-communal level, produced by INSEE: contains the number of active persons aged 15-64 years: https://www.insee.fr/fr/statistiques/2028672 | 2015 census data  Date received: 2023-02-21  FDep 2015 and components at the communal level: file directly transmitted by email by the producer (INSERM-CEPIDC) |
| Proportion of high school graduates in the unschooled population aged ≥15 years |  |  |
| Proportion of manual workers in the active population aged 15–64 years |  |  |
| Unemployment rate among the active population aged 15–64 years |  |  |
| FDep (French Deprivation Index) |  |  |
| Proportion of overcrowded households | Source: 2010 census data, by commune  File directly transmitted by email by the producer (INSEE) | Source: 2016 census data, by commune  File directly transmitted by email by the producer (INSEE) |
| Population density level | Same dataset for both periods  4-level communal density grid constructed from the 2010 population census data, projected onto the communal division in force on January 1, 2017, https://www.insee.fr/fr/information/2114627 | |

# Definition

**Definition of overcrowded household** by the INSEE - French National Institute of Statistics and Economics Studies (https://www.insee.fr/fr/metadonnees/definition/c1236, accessed Feb. 10 2025):

The theoretically necessary number of rooms in a household (i.e. the “norm”) is calculated as follows:

- a living room for the household;
- one room for each couple;
- one room for each person aged 19 or over not living as a couple in the household;
- for people under 19 years old, one room for two people if they are of the same sex or under 7 years old, otherwise, one room per person.

A household is defined as **overcrowded** if it has fewer rooms than this norm.

# ZIP code-level sociodemographic contextual variables

## Distribution

*Table 2. Characteristics of the ZIP code-level socioeconomic variables, in metropolitan France, 2008*–*2019, N=5534 ZIP codes.*

|  | [ALL] | 2008–2013 | 2014–2019 | P-value |
| --- | --- | --- | --- | --- |
| Proportion of overcrowded households, Median [25th;75th] | 1.79 [1.13;2.90] | 1.87 [1.19;2.98] | 1.71 [1.08;2.82] | <0.001* |
| Unemployment rate among the active population aged 15–64 years, Median [25th;75th] | 10.5 [8.33;13.3] | 9.33 [7.42;11.8] | 11.7 [9.43;14.4] | <0.001* |
| Median household income per consumption unit, Median [25th;75th] | 18,796 [16,952;21,193] | 17,663 [16,064;19,932] | 19,739 [18,154;22,256] | <0.001* |
| French Deprivation Index, Median [25th;75th] | 0.42 [-0.42;1.09] | 0.41 [-0.35;1.02] | 0.43 [-0.50;1.18] | 0.048* |
| Proportion of manual workers in the active population aged 15–64 years, Median [25th;75th] | 25.7 [20.0;31.6] | 26.6 [20.7;32.5] | 24.9 [19.3;30.7] | <0.001* |
| Proportion of high school graduates in the unschooled population aged ≥15 years, Median [25th;75th] | 37.3 [31.5;44.2] | 34.5 [29.2;41.2] | 39.9 [34.2;46.5] | <0.001* |
| Population density level in 2010, N (%): |  |  |  | NA^£^ |
| Low | 6672 (60.3%) | 3336 (60.3%) | 3336 (60.3%) |  |
| Medium | 2986 (27.0%) | 1493 (27.0%) | 1493 (27.0%) |  |
| High | 1410 (12.7%) | 705 (12.7%) | 705 (12.7%) |  |

* Kruskall-Wallis test

^£^ Comparison not applicable: the same population density level is used for both periods.

Figure 1. Distribution of the studied ZIP code-level socioeconomic variables, 2008–2019, metropolitan France. NB: there are two observations for each variable, in each ZIP code: one for the 2008–2013 period, one for the 2014–2019 period. (A) the original set of variables; (B) The set of variables used for modelling: the four variables composing the French Deprivation Index (% unemployment, % of overcrowded households, % of workers, % of high-school graduates) are log-transformed.

| **(A)** | | 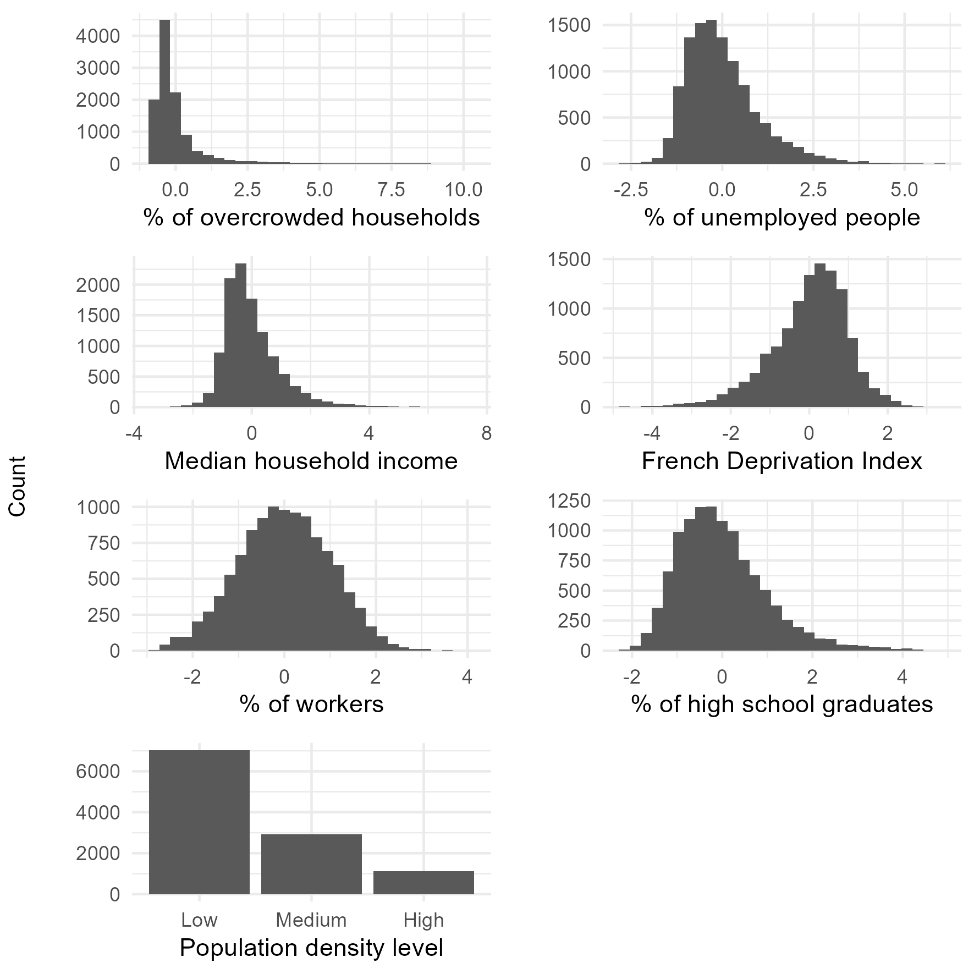 |
| --- | --- | --- |
| **(B)** | 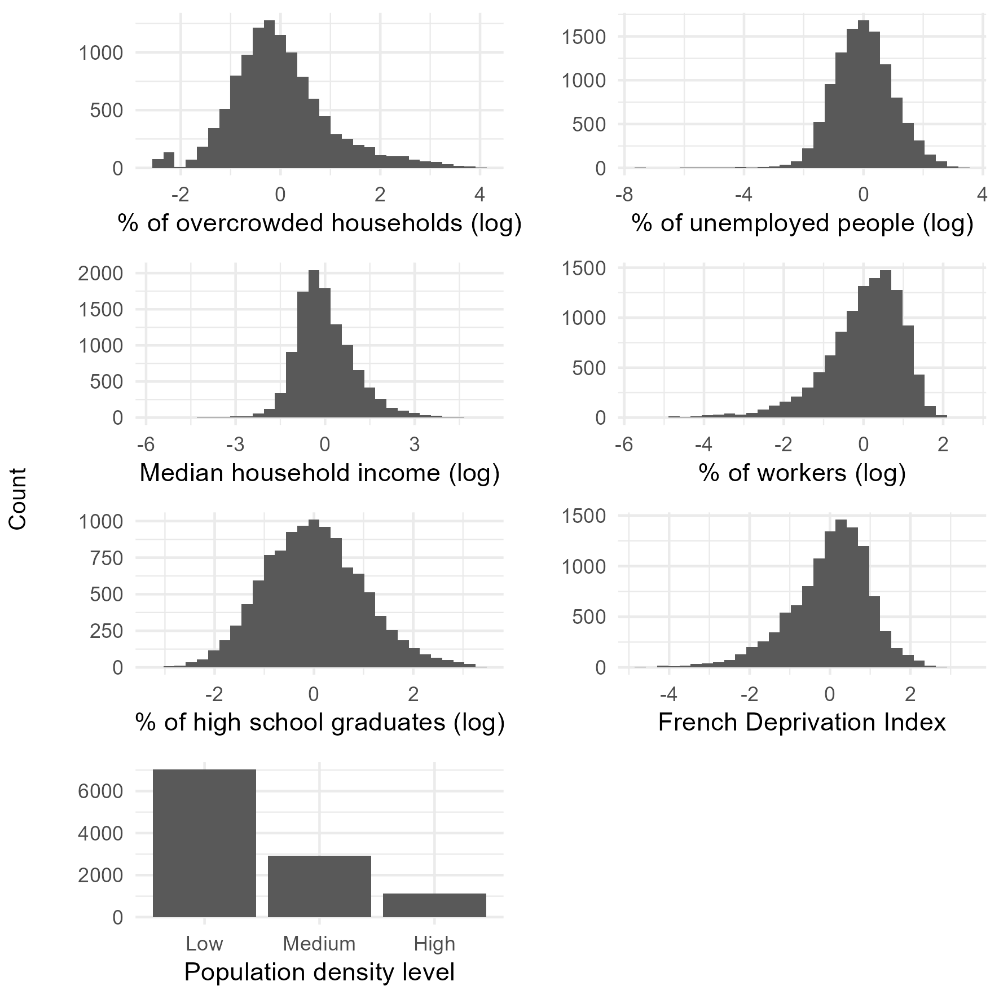 | |

## Correlation between the continuous explanatory variables

The continuous covariate were log-transformed to achieve a more symmetric distribution, except for the FDEP.

The Pearson correlation coefficient between the (log-transformed) continuous covariates at the ZIP code level varied between -0.92 and 0.9 and was always statistically significant (P<0.001), see the correlation matrix in Figure 2.

Figure 2. Correlation matrix of the continuous explanatory variables. (A) Representing distribution plots and bivariate scatterplots; (B) Ordering the variables using a hierarchical clustering analysis, with the “average” agglomeration method. The rectangles outline the clusters obtained when partitioning the dataset of variables into three groups.

| **(A)** | | 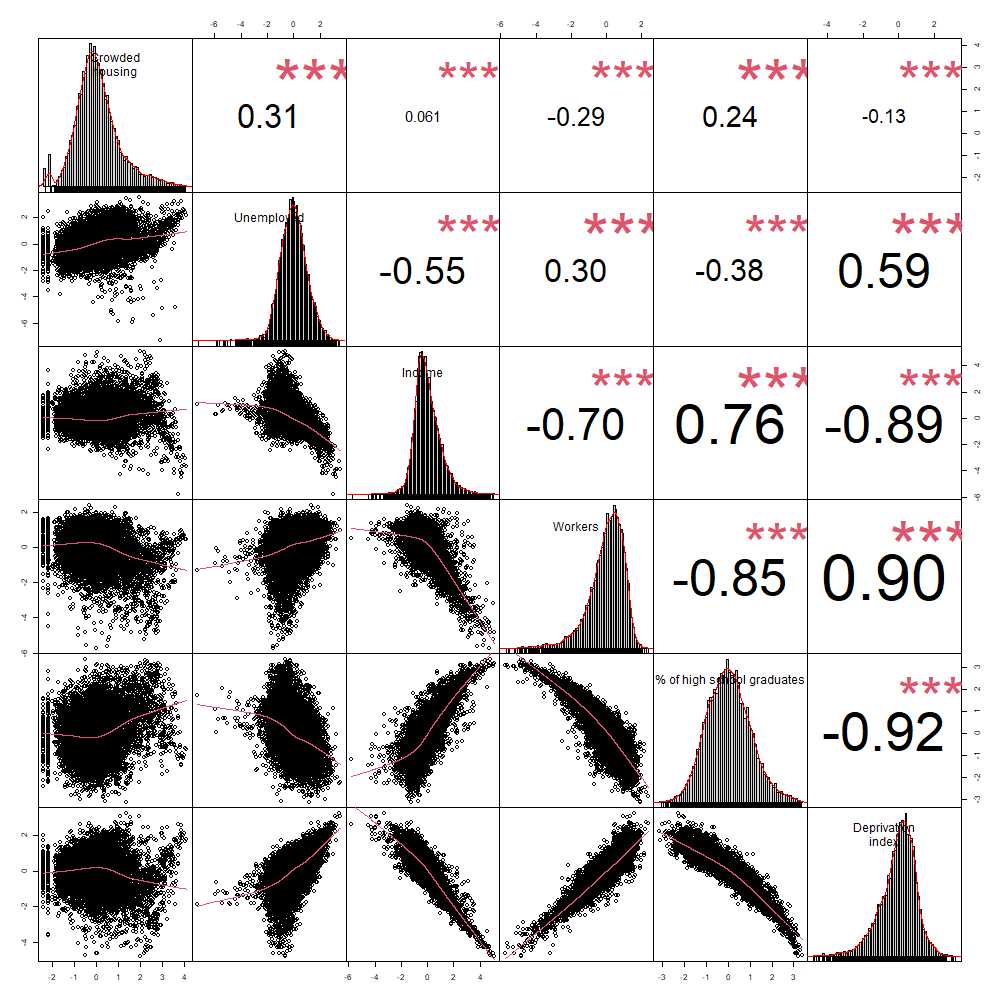 |
| --- | --- | --- |
| **(B)** | 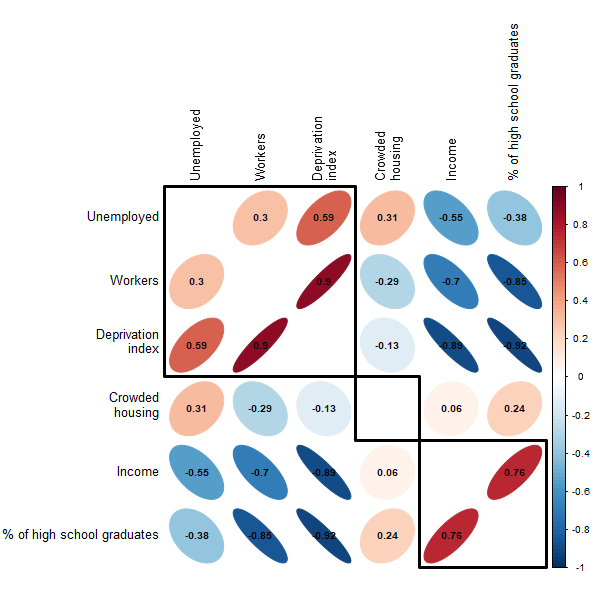 | |

## Association between density levels and the continuous variables

The population density level was significantly associated with all the continuous explanatory variables (Fisher’s test P < 0.001). For example, denser ZIP codes had a smaller proportion of workers and a greater proportion of high school diploma (see Figure 3).

Figure 3. Distribution of the ZIP code-level continuous explanatory variables stratified by population density level. All variables but the French Deprivation Index (FDep) were log-transformed.


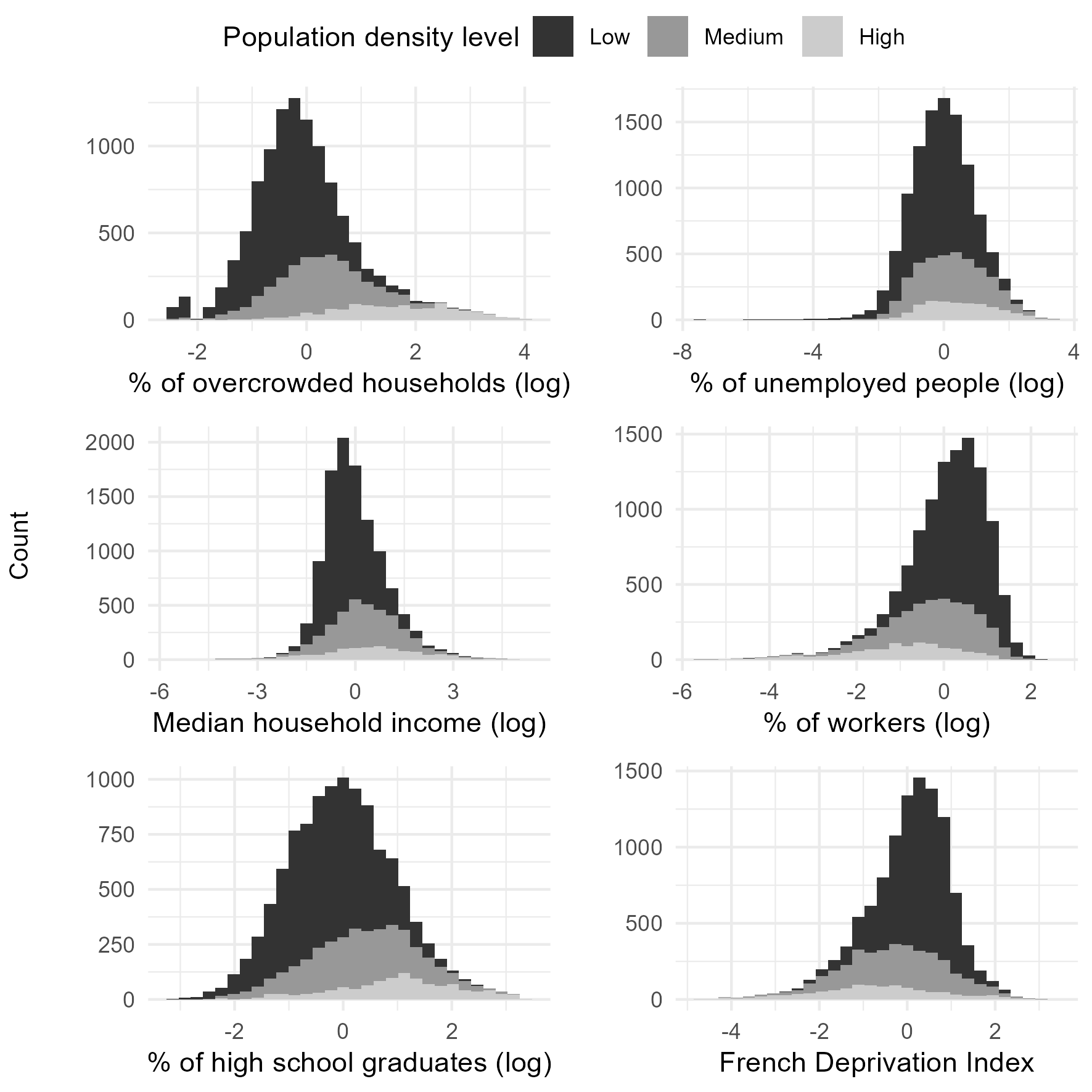


# Spatial model: supplementary results

## Random effects with the BYM2 model

As visible in Figure 4, the spatial random effects are shrinked towards zero in the multivariable model. In other words, the four variables of this model have “explained” some of the variability of the random effect. This is particularly visible for Paris area (squared top-right insert), where ZIP codes in red (having higher relative risks) go back to pale yellow (average relative risks). Yet, as discussed in the main text, the random effect comprises a mix of territorial discrepancies in incidences AND completeness of the notification rates, since practitioners fail to report all tuberculosis cases as normally required, with high territorial variability [1]. The completeness of the notification rates has not been re-evaluated since Girard et al study in 2010 [1]. Therefore, one cannot go further in the qualification and quantification of territorial discrepancies of TB notification rates.

Figure 4. Map of the spatial random effects, $U_{i}$, in the empty model (A) and the multivariable model (B).


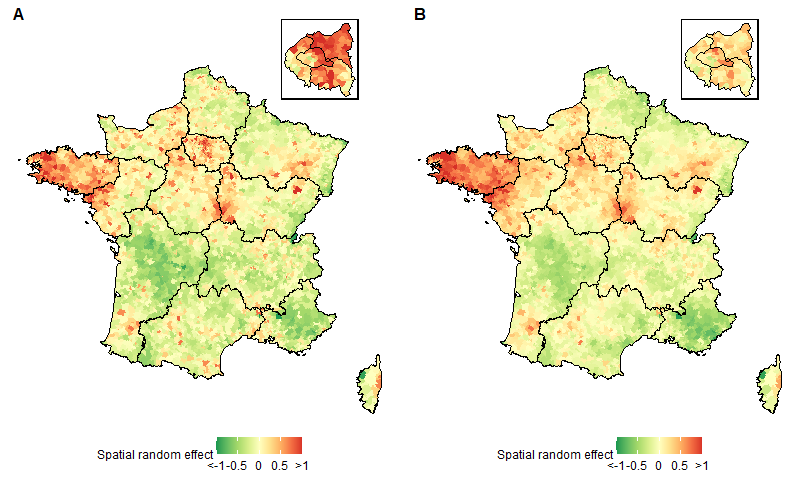


## Alternative spatial model: the Leroux model

We ran the multivariable model with an alternative specification of the spatial random effect, namely the Leroux model, as a sensitivity analysis [2].

### Description of the Leroux model

Following the explanation in [3], in the Leroux model, the spatial random effect, b, is assumed to follow a normal distribution with mean zero and covariance matrix:

$$\mathrm{Var}\left( \mathbf{b} | \tau_{b},\phi\right)=\tau_{b}^{-1}\left( \left( 1-\phi\right)\mathbf{I}+\phi\mathbf{Q} \right)^{-1}$$

Where $\phi\in\left[ 0, 1 \right]$ denotes a mixing parameter. The model reduces to pure overdispersion if $\phi=0$, and to the Besag model when $\phi=1$. The conditional expectation of $b_{i}$, given all other random effects, results as a weighted average of the zero-mean unstructured model and the mean value of the Besag model. The conditional variance is the weighted average of $\frac{1}{\tau_{b}}$ and $\frac{1}{{(\tau}_{b}.n_{\delta_{i}})}$, where $n_{\delta_{i}}$ is the number of neighboring areas that area *i* has.

### Comparison of the BYM2 and Leroux model outputs

The maps of the estimated standardized notification rates and spatial random effects appear very similar between both models (Figure 5). Figure 6 further illustrates how close the spatial random effect distributions are between the BYM2 and Leroux multivariable models. Finally, Figure 7 displays the association between each explanatory variable and TB standardized notification rates, in both models: the estimated effects of both models greatly overlap. This similarity is also visible in Table 3, which summarizes these effects with inter-decile standardized rate ratio (IdRR). Finally, the BYM2 and Leroux specification yield equivalent goodness-of-fit statistics, may it be in the model without explanatory variable or in the multivariable model (Table 4).

Figure 5. Estimated standardized notification rate (SNR) and spatial random field $U_{i}$ in the multivariable model. The spatial random effect at the ZIP code level are modelled with a BYM2 model (left hand side, reference model) or a Leroux model (right hand side, alternative model tested as sensitivity analysis).


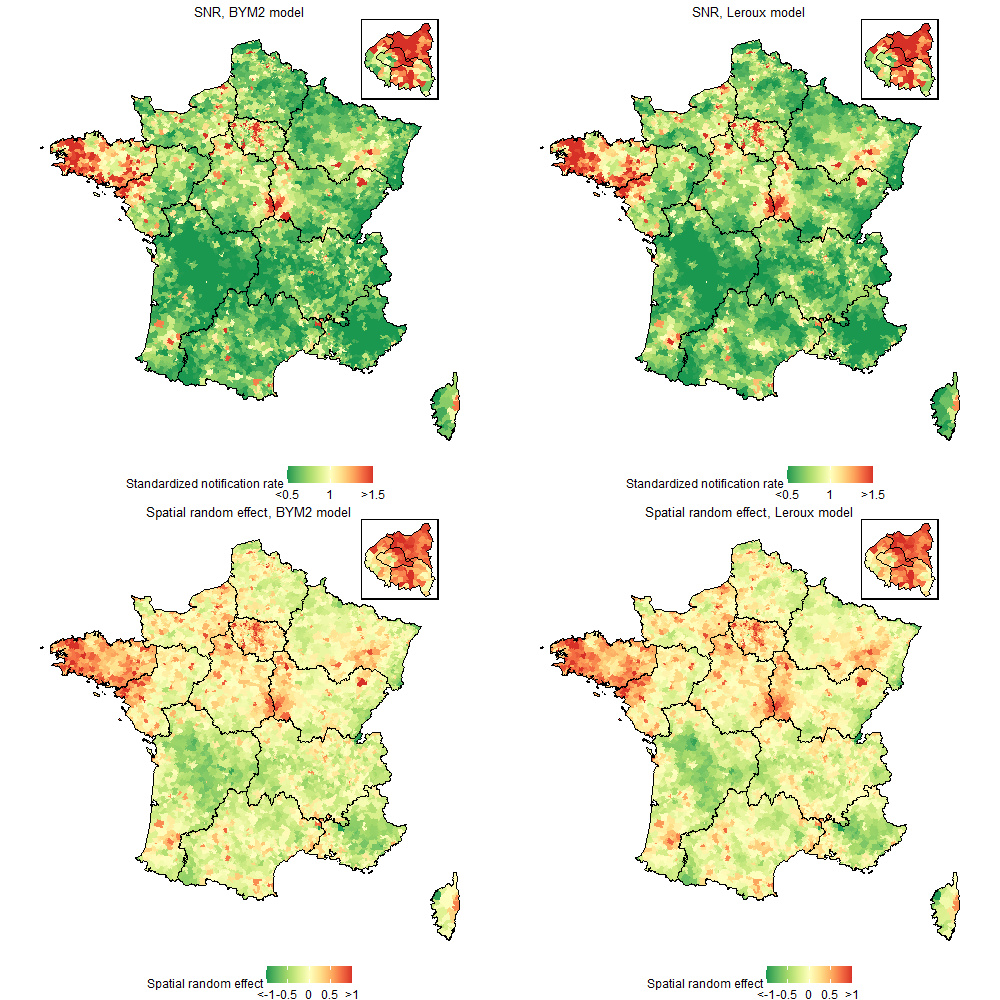


Figure 6. Distribution of the spatial random effect U_i in the multivariable reference model (BYM2) and in the Leroux model.


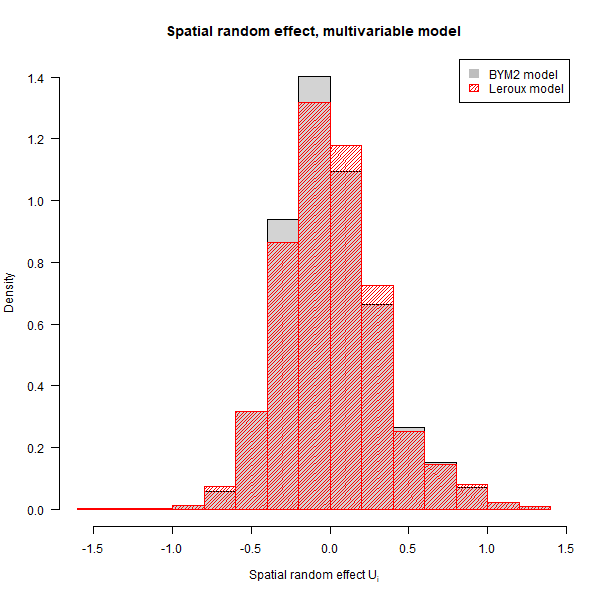


Figure 7. Association between the explanatory variables and the standardized TB notification rates in the multivariable BYM2 model (black) and Leroux model (red).


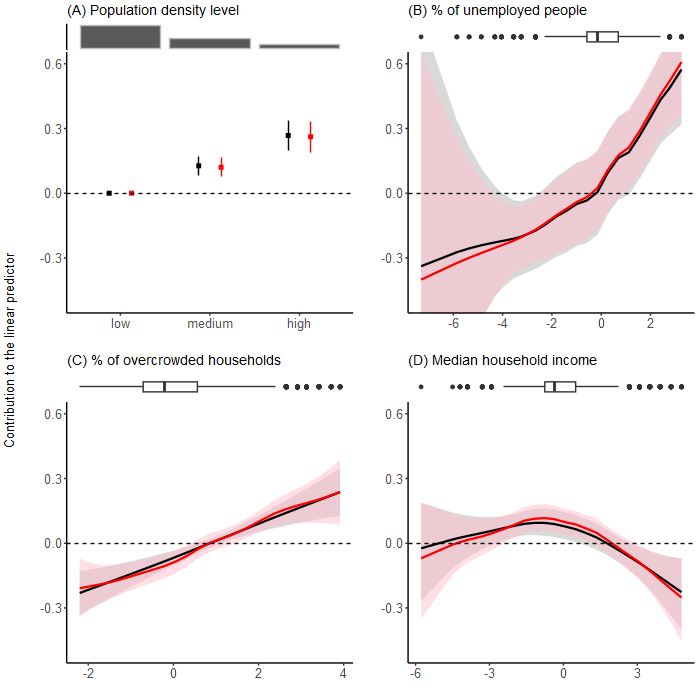


Table 3. Inter-decile standardized rate ratio, for each variable in the multivariable model, with the BYM2 and Leroux models.

| Variable | IdRR BYM2  (95% CrI) | IdRR Leroux  (95% CrI) |
| --- | --- | --- |
| Population density level | 1.30 (1.21; 1.38) | 1.31 (1.21; 1.42) |
| % of unemployed people | 1.28 (1.19; 1.37) | 1.28 (1.19; 1.37) |
| % of overcrowded households | 1.19 (1.11; 1.28) | 1.24 (1.14; 1.34) |
| Median household income | 0.93 (0.89; 0.99) | 0.94 (0.90; 0.99) |

Table 4. Comparison of the BYM2 and Leroux models goodness of fit statistics. Deviance Information Criterion (DIC), Watanabe–Akaike information criterion (WAIC), and ``leave-one-out'' predictive measures of fit : CPO $(Prob\left( y_{i} \right|y_{\left\{ -i \right\}}))$ and PIT $\left( Prob\left( y_{i}^{new}\leq y_{i} \right|y_{\left\{ -i \right\}} \right)$.

| Model | DIC | WAIC | CPO | PIT |
| --- | --- | --- | --- | --- |
| Empty BYM2 | 36,230 | 36,831 | 0.29 | 0.62 |
| Empty Leroux | 36,418 | 37,029 | 0.28 | 0.61 |
| Multivariate BYM2 | 35,961 | 36,577 | 0.30 | 0.64 |
| Multivariate Leroux | 36,054 | 36,661 | 0.30 | 0.63 |

# Imputation of missing information of tuberculosis cases

There were missing values in the variables used for defining strata during the calculation of the expected TB counts in each ZIP code. Therefore, we imputed those missing values before proceeding to the calculation of the expected values using three different imputation methods, to ensure that the results were not biased by the choice of the imputation method.

Table 5. Number of missing values for each variable used for stratifying the data to construct expected values in each ZIP code, out of 55,330 observations.

| Variable | Number of missing values (%) |
| --- | --- |
| Age | 42 (0.1 %) |
| Sex (male/female) | 348 (0.6 %) |
| Immigration status (immigrant/native) | 3913 (7.1 %) |
| Type of housing (individual housing, prison, other communal housing) | 7409 (13.4 %) |

## Imputation methods

### Reference method: the k nearest neighbors (kNN) imputation method

In our central analysis, we used the kNN imputation method implemented in the R package VIM [4].

First we extracted the dataset consisting of the standardized age (mean 0, standard deviation 1), sex (Male/Female), immigration status (immigrant/native), “lives in a communal housing” (Yes/No), “homelessness” (Yes/No), and period (2008–2013 and 2014–2019).

Secondly, we applied the kNN algorithm to impute the missing values in this dataset.

Third, only for the cases with “lives in a communal housing” equal to Yes (whether imputed or not), we imputed the variable “type of communal housing” (Prison/Other communal housing) by running the kNN algorithm on the formerly imputed dataset enriched with the variable “lives in a communal housing”.

Fourth, for the cases with “lives in a communal housing” equal to No (whether imputed or not), “type of communal housing” was set to “Individual housing”.

### Alternative method 1: imputation with Random Forests

In a first sensitivity analysis, we used the Random Forest imputation method implemented in the R package missForest [5].

First we extracted the dataset consisting of the standardized age (mean 0, standard deviation 1), sex (Male/Female), immigration status (immigrant/native), “lives in a communal housing” (Yes/No), “homelessness” (Yes/No), and period (2008–2013 and 2014–2019).

Secondly, we applied the missForest algorithm to impute the missing values in this dataset.

Third, only for the cases with “lives in a communal housing” equal to Yes (whether imputed or not), we imputed the variable “type of communal housing” (Prison/Other communal housing) by running the missForest algorithm on the formerly imputed dataset enriched with the variable “lives in a communal housing”.

Fourth, for the cases with “lives in a communal housing” equal to No (whether imputed or not), “type of communal housing” was set to “Individual housing”.

### Alternative method 2: proportional distribution of missing values between the closest strata

In a second sensitivity analysis method, we used a very simple algorithm to distribute missing cases between the closest categories of cases.

First, we calculated the observed number of tuberculosis cases in metropolitan France, stratified by period (2008-2013, 2014-2019), sex (male, female), age group (0-14, 15-24, 25-44, 45-64, ≥65 years-old), immigration status (immigrant, non-immigrant) and housing type (individual housing, prison, other communal housing).

Then, missing values of age group, sex, immigration status and housing category were imputed using the distribution observed in non-missing values of cases belonging to the closest strata. For example, if sex information was missing for three cases that belonged to the “2008-2013 period, 0-14 years-old, immigrant, individual housing” stratum, and if the proportion of female cases was 0.4 for this stratum, then the three cases were dispatched as follows: 3x0.4=1.2 females and 3x0.6=1.8 males. If ‘sex’ and ‘immigration status’ were missing for 4 cases belonging to the “2008-2013 period, 0-14 years-old, individual housing” stratum, and if the proportion of Female-Immigrant was 0.1, Male-Immigrant 0.2, Female-Native 0.3, and Male-Native 0.4, then the 4 cases with missing values were dispatched as follows : 4x0.1=0.4 Female-Immigrant case, 4x0.2=0.8 Male-Immigrant case, 4x0.3= 1.2 Female-Native case and 4x0.4=1.6 Male-Native case. Note that the results of each imputation step is not used in the next imputation step.

After the imputation step, the number of tuberculosis cases in each stratum was not necessarily round, and the sum of cases over all strata was equal to the total number of tuberculosis cases over metropolitan France.

## Imputation results

Figure 8. The standardized notification rate (SNR, top panels) and the spatial random effect (U_i_, bottom panels), obtained with the multivariable model. Left column: k nearest neighbors (kNN) imputation method (central analysis); center column: random forest (RF) imputation method; right column: simple proportional distribution of missing values between similar strata.


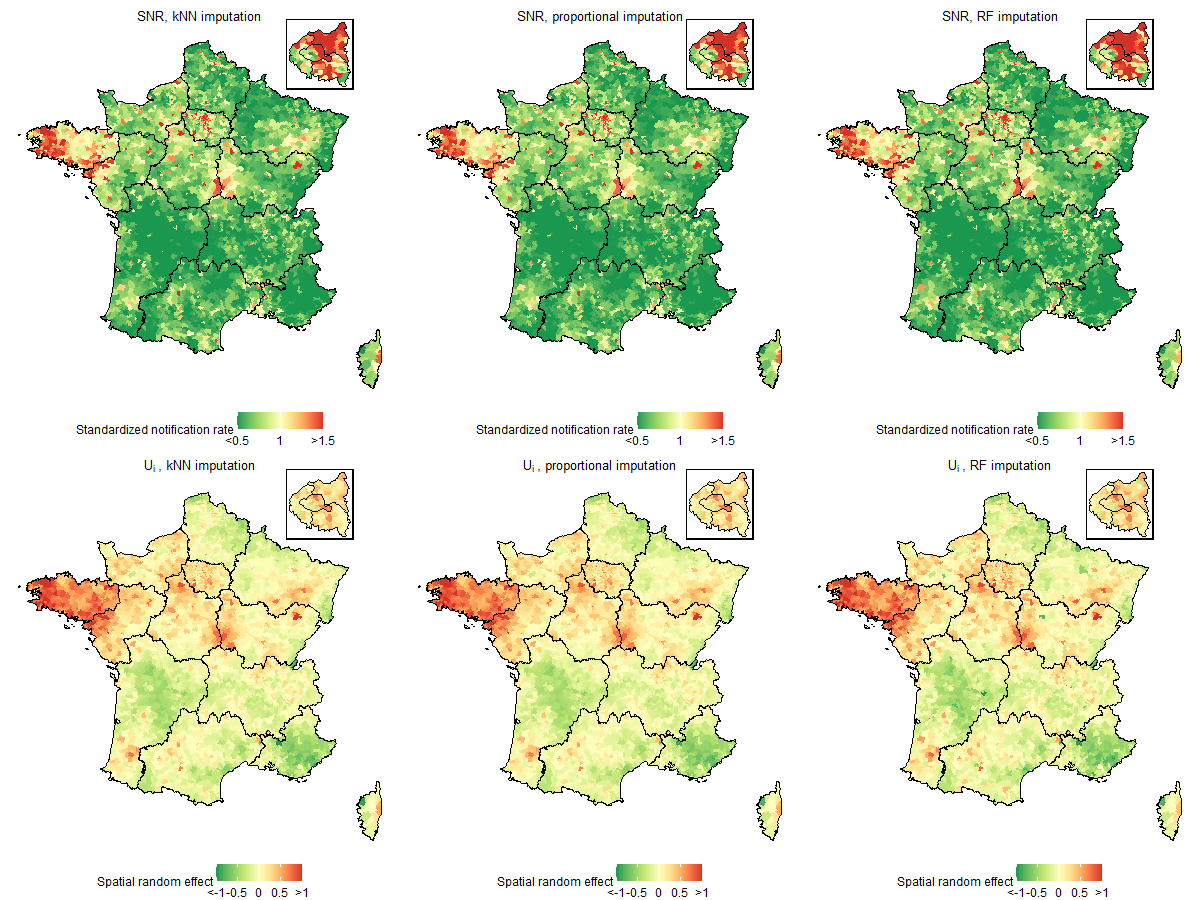


Figure 9. Contribution of each explanatory variable to the linear predictor (log of the standardized notification rate), in the multivariable model. Black: imputation of missing values with the k nearest neighbors’ method (central analysis); green: imputation with the random forest method; red: simple proportional distribution of missing values between similar strata.


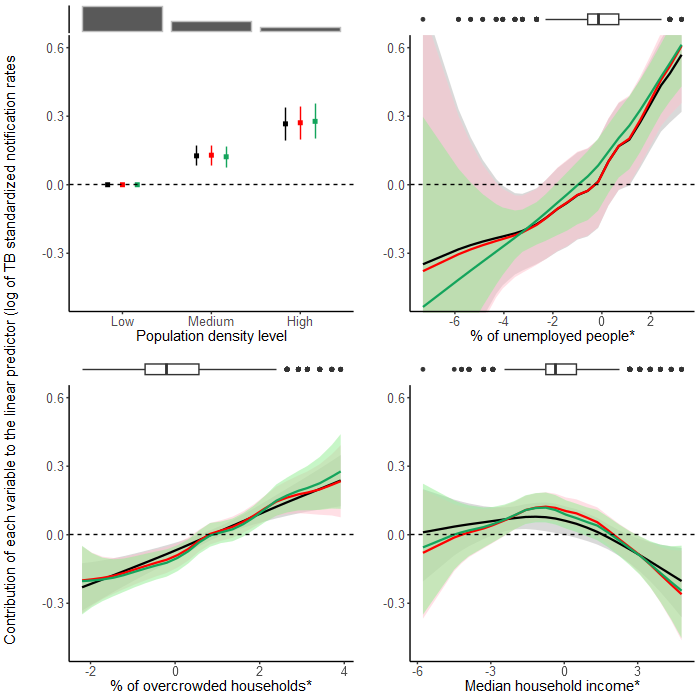


Table 6. Inter-decile standardized rate ratio (IdRR) for each variable, in the multivariable model and associated 95% credible intervals (95% CrI) for three different imputation methods of missing values. Left column: k nearest neighbors (kNN) imputation method (central analysis); center column: random forest (RF) imputation method; right column: simple pro rata distribution of missing values between similar strata.

| Variable | IdRR kNN imputation (95% CrI) | IdRR pro rata imputation (95% CrI) | IdRR RF imputation (95% CrI) |
| --- | --- | --- | --- |
| Population density level | 1.30 (1.21, 1.38) | 1.32 (1.25, 1.40) | 1.32 (1.22, 1.41) |
| % of unemployed people | 1.28 (1.19, 1.37) | 1.28 (1.20, 1.37) | 1.28 (1.20, 1.37) |
| % of overcrowded households | 1.19 (1.11, 1.28) | 1.21 (1.10, 1.30) | 1.20 (1.10, 1.30) |
| Median household income | 0.93 (0.89, 0.99) | 0.94 (0.88, 0.99) | 0.93 (0.86, 0.99) |

# References

1. Girard D, Antoine D, Che D. Epidemiology of pulmonary tuberculosis in France. Can the hospital discharge database be a reliable source of information? Med Mal Infect. 2014;44(11-12):509-514.

2. Leroux BG, Lei X, Breslow N: Estimation of Disease Rates in Small Areas: A new Mixed Model for Spatial Dependence. In: Statistical Models in Epidemiology, the Environment, and Clinical Trials: 2000// 2000; New York, NY: Springer New York; 2000: 179-191.

3. Riebler A, Sørbye SH, Simpson D, Rue H. An intuitive Bayesian spatial model for disease mapping that accounts for scaling. Statistical Methods in Medical Research. 2016;25(4):1145-1165.

4. Kowarik A, Templ M. Imputation with the R Package VIM. Journal of Statistical Software. 2016;74(7):1-16.

5. Stekhoven DJ, Buhlmann P. MissForest--non-parametric missing value imputation for mixed-type data. Bioinformatics. 2012;28(1):112-118.
